# Beyond Traditional Medicine: EVs-Loaded Hydrogels as a Game Changer in Disease Therapeutics

**DOI:** 10.3390/gels10030162

**Published:** 2024-02-21

**Authors:** Shutong Du, Xiaohu Zhou, Bo Zheng

**Affiliations:** Institute for Cell Analysis, Shenzhen Bay Laboratory, Shenzhen 518132, China; dushutong@szbl.ac.cn (S.D.); zhouxh@szbl.ac.cn (X.Z.)

**Keywords:** extracellular vesicles, hydrogel, cell-free therapy

## Abstract

Extracellular vesicles (EVs), especially exosomes, have shown great therapeutic potential in the treatment of diseases, as they can target cells or tissues. However, the therapeutic effect of EVs is limited due to the susceptibility of EVs to immune system clearance during transport in vivo. Hydrogels have become an ideal delivery platform for EVs due to their good biocompatibility and porous structure. This article reviews the preparation and application of EVs-loaded hydrogels as a cell-free therapy strategy in the treatment of diseases. The article also discusses the challenges and future outlook of EVs-loaded hydrogels.

## 1. Introduction

Traditional cell therapies like stem cell transplantation hold tremendous potential for treating diseases such as myocardial infarction [1,2]. However, these cell therapies may encounter various limitations, including low stability in the storage and transportation of living cells, potential risks of tumorigenicity and immunogenicity, and high cost of treatment [3,4,5,6]. To overcome these limitations, cell-free therapy (CFT) provides an alternate exciting approach [7,8,9,10,11] in which bioactive molecules such as protein, mRNA, and miRNA can perform the primary functions of cells [12,13].

The bioactive molecules for CFT can be extracted from cell culture media [14,15]. During the cell cultivation process, cells release extracellular vesicles (EVs) into the surrounding environment. EVs are sub-micron-sized particles with a phospholipid membrane and contain molecules from cells, including proteins, nucleic acids, and soluble small molecules [16,17]. EVs play a crucial role in CFT [18]. The three main types of EVs are exosomes (40–160 nm), microvesicles (100–1000 nm), and apoptotic bodies (50–5000 nm) [19,20]. Researchers have discovered that mesenchymal stem cells (MSCs) secrete a form of EVs containing miRNA associated with tumors [21]. These EVs can influence the behavior of tumor cells [22] and have potential applications in cancer treatment [23].

However, EVs are subject to rapid clearance, which presents an obstacle for the application of the therapeutic EVs in CFT. For example, EVs isolated from melanoma cells (B16BL6) are rapidly cleared within approximately 2 min after intravenous injection in mice [24]. During the transportation process in the bloodstream, EVs are easily cleared by macrophages [25] and tend to accumulate in the liver, spleen, and lungs, rather than at the lesion sites [26]. To solve this problem, researchers have used hydrogels to protect EVs from the clearance of immune cells [25], successfully deliver EVs and persistently keep them at lesion sites [27]. EVs-loaded hydrogel offers several advantages in disease treatment, including a high loading rate of EVs, sustained release of EVs, and cryopreservation stability [28].

In this work, we review the production and applications of EVs-loaded hydrogels in disease therapy. We will describe the detailed process of synthesizing EVs-loaded hydrogels, the critical biomolecules that contribute to their therapeutic efficacy, and their specific applications in treating various diseases, such as myocardial infarction, intervertebral disc degeneration, osteoarthritis, bone deficiency, diabetic ulcers, and nerve injury. Additionally, we critically assess the significance and potential limitations of using these hydrogels in disease therapy, offering an insightful evaluation of their role in advancing medical treatments.

## 2. Synthesis of EVs-Based Hydrogels

**Figure 1:**
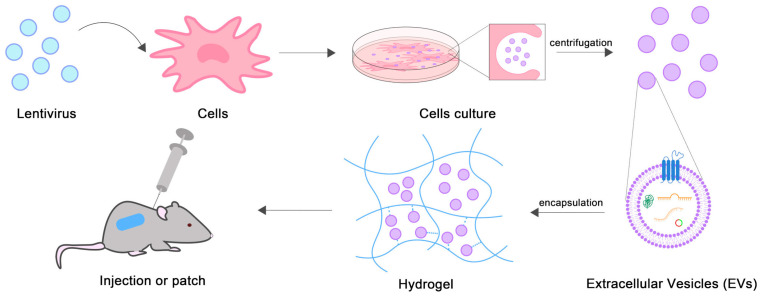
Schematic diagram of EVs-loaded hydrogels synthesis.

The synthesis of EVs-based hydrogels is shown in Figure 1. First, EVs are isolated, typically by ultracentrifugation from cell culture medium supernatants. Then, the isolated EVs are assembled into injectable hydrogels or hydrogel patches by in situ polymerization or adsorption.

### 2.1. Acquisition of EVs

EVs can be classified into native EVs, engineered cell EVs, and post-modified EVs based on the source of their components [29]. There are three major pre-treatment methods for the acquisition of therapeutic EVs. The most primitive method is the isolation of EVs directly from the source cells without any means of intervention [30]. For example, MSCs are able to differentiate into a variety of cell types, including bone, cartilage, fat, etc. [1,31,32]. The therapeutic effect of EVs isolated from MSCs is similar to that from MSCs transplantation [30]. Xia et al. [33] isolated EVs derived from umbilical cord mesenchymal stem cells (UCMSCs) for bone repair. Sun et al. [34] isolated EVs from bone marrow mesenchymal stem cells (BMSCs) to promote epiphyseal plate damage restoration.

Another common approach is to induce parental cells by hypoxia to obtain engineered cell EVs enriched with specific biomolecules [35]. Researchers have shown that the gene regulation patterns of cells are significantly different under hypoxia induction [36], and there are hypoxia activation and repression genes [37,38,39]. Bai et al. [40] used hypoxia-induced BMSCs to isolate EVs for the treatment of myocardial infarction. Zuo et al. [41] isolated EVs from hypoxia-induced BMSCs for cartilage regeneration. The isolation of EVs after lentiviral transfection of cells is also a method of interest to researchers [42]. Zhao et al. [43] incubated BMSCs with retroviruses, and the resulting EVs overexpressed miR-29b-3p, reducing scarring during wound healing. Li et al. [44] transfected UCMSCs with pCDH virus, up-regulated the expression of 5′-nucleotidase (CD73) in EVs, and attenuated inflammation after spinal injury.

In addition, the unique protein- and carbohydrate-decorated surface of EVs makes EVs a promising natural nanocarrier [45,46]. Exogenous RNA, proteins, and small molecules can be packaged in EVs by methods such as co-incubation, electroporation, freeze-thawing, ultrasound, extrusion, etc. [47,48,49]. As a nanocarrier, EVs can protect the molecules of interest from immune clearance, facilitate the molecules to cross biological barriers, and enhance the binding specificity of the molecules to the target tissues [50].

### 2.2. Package of EVs on Hydrogels

EVs can be loaded into hydrogels through in situ polymerization or adsorption. The interactions between the polymer backbone of hydrogels and EVs include van der Waals forces, hydrogen bonding, and electrostatic interaction, which enhance the stability of the EVs in the hydrogel and thus prolong the residence time of the EVs in vivo [51]. The in situ polymerization method involves the co-mixing of a gel precursor solution and an EVs solution. The polymerization conditions must be mild, e.g., 365 nm UV irradiation for 1 min [52], and polymerization at room temperature [53] or 37 °C [54]. Alternatively, hydrogels can also be prepared first, and then mixed with the EVs solution to allow the adsorption of EVs into hydrogels [55]. The most often used hydrogel materials for EVs delivery include calcium alginate (Ca-Alg) [53,56], arginine-glycine-aspartate (RGD) [57,58,59], hyaluronic acid (HA) [55,60,61,62], chitosan (CS) [54,63,64,65,66], and gelatin methacrylate (GelMA) [34,52,67,68]. The synthesis of the hydrogel has been well documented in many reviews [69,70,71,72,73,74,75,76,77,78,79], and is not described in the current work.

Compared with the interaction between EVs and the polymer backbone of hydrogels in the common physical encapsulation, the integrin present on the surface of the EVs’ membrane [80] has much stronger binding affinity with RGD peptides [81], which can increase the stability of EVs in the hydrogels presenting RGD in the polymer backbone. As a result, the functional lifetime of the EVs can be increased [59]. In addition, the dynamic hydrogen bonding between the DA of the dopamine-grafted gelatin (GelDA) gel and the surface amines of EVs can promote the attachment of EVs to the hydrogel network, leading to the development of heat-sensitive or ROS-sensitive composite gels for precise EVs release in therapy [82].

## 3. Bioactive Molecules in EVs Used for Disease Treatment

### 3.1. Protein

In EVs, most proteins are secreted by parent cells in a soluble form and embedded in the surface phospholipid bilayer [83], and applied to intercellular communication [84]. The lack of relevant proteins may lead to unregulated intercellular signal transduction or obstacles in transportation, thereby triggering numerous diseases. Treatment of related diseases often requires restoring protein function [85]. Biological functional enzymes or therapeutic proteins are susceptible to protein degradation and poor cellular uptake during transport, and EVs are ideal delivery vehicles for proteins [86].

### 3.2. mRNA

The safety and conversion efficiency of mRNA through chemical modification in vitro synthesis were demonstrated in the widespread use of mRNA as a vaccine in the 2019 Novel Coronavirus Disease (COVID-19) pandemic [87,88]. This successful case has sparked widespread interest in using mRNA in both the application of vaccines and other medical needs [89,90]. mRNA can be encapsulated into isolated EVs and then endocytosed into recipient cells to express targeted proteins [91].

### 3.3. miRNA

A key active ingredient in achieving therapeutic effects in EVs is miRNA [92]. miRNA is a 19–24 nucleotide long non-coding RNA that regulates gene expression by targeting the 3′-untranslated region (3′-UTR) of mRNA [93]. Various distinct cellular expression processes can be regulated by a single type of miRNA [94]. In different forms of EVs secreted by various cells, or even one type of cells, there may be different carried miRNAs, exerting distinct regulatory effects. Compared to drugs that act on a single gene, miRNA possesses a stronger biomolecule capability to selectively regulate multiple genes [95]. Importantly, researchers have found that, to enhance its therapeutic effects, it is possible to engineer the enrichment or design modifications of miRNAs contained in EVs through interventions on parent cells such as lentiviral transduction [29,43,96].

### 3.4. circRNA

In some situations, miRNAs may be degraded by RNA exonucleases. In contrast, circular RNA (circRNA), a covalently closed, single-stranded, circular RNA [97], exhibits excellent environmental resistance and stability [98,99]. In the absence of coding for proteins, circRNA regulates the behaviors of cells by modulating the expression of miRNAs or downstream proteins [100]. CircRNA has been shown to play an important role in a variety of cancers [101]. CircRNAs are predominantly located in the nucleus and can be packaged in EVs for transport in circulation [102].

### 3.5. Antagomir

Antagomirs, sometimes referred to as anti-miRNA [103], are microRNA inhibitors with a chemical modification of homologous miRNA. The modification makes antagomirs more resistant to degradation and more effective in inhibiting miRNA expression [104,105]. The difference between antagomir and normal RNA is the complete 2′-O-methylation of three parts, namely the ribose part, the thiosulfate bond part, and the 3′-end cholesterol part [105,106,107]. Antagomir has shown cutting-edge promise in treating fluorescent tumors, as it does not act on tumor cells in a cell-by-cell toxic manner, but rather prevents them from initiating metastasis [108].

## 4. The Application of EVs-Loaded Hydrogels in Disease Treatment

### 4.1. Myocadial Infarction

The non-proliferative nature of adult cardiomyocytes leads to the fibrosis of damaged myocardium [109]. Researchers have pointed out that EVs secreted by mesenchymal stem cells (MSCs) [110], cardiac progenitor cells (CPCs) [111], and induced pluripotent stem cells (iPSCs) [112,113] carry important molecules in heart-specific processes, which can promote angiogenesis, inhibit apoptosis and fibrosis, reduce cardiac ischemic injury [114,115], and protect the cardiac function of ischemic heart disease [116] while avoiding the tumorigenicity and high tendency of arrhythmia brought about by stem cell therapy or cardiomyocyte therapy.

**Figure 2:**
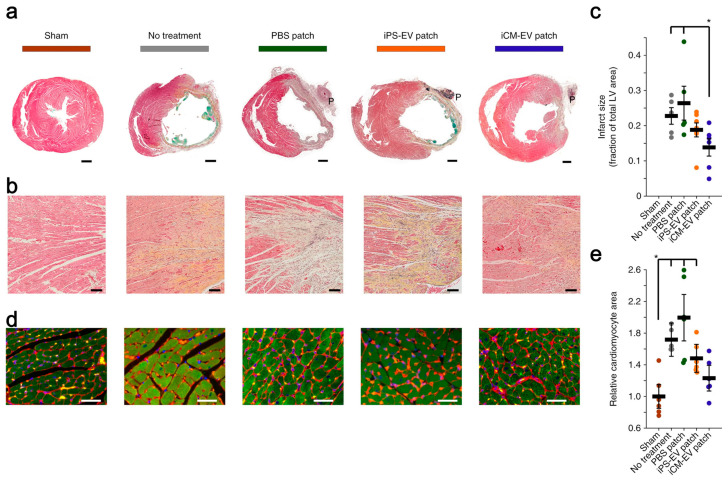
iCM-EVs-collagen gelfoam patches reduced infarct size and pathological hypertrophy. (**a**) Transverse cardiac sections after treatment with patches. Scale bars: 1 mm; (**b**) high-power images of the infarct border zone. Scale bars: 100 μm; (**c**) infarct size as a percentage of the total area of the left ventricle (LV). * *p* < 0.05 by two-tailed *t*-test; (**d**) sections were stained with wheat-germ agglutinin (red), troponin (green), and DAPI (blue). Scale bars: 50 μm; (**e**) relative cardiomyocyte area quantified. * *p* < 0.05 by two-tailed *t*-test. [117].

Gordana et al. [117] assembled EVs secreted by induced cardiomyocytes (iCMs) into the hydrogel patches to form cardiac patches, and this process was achieved by the in situ polymerization of collagen within a gelfoam mesh at 37 °C. The patches can continuously release iCM-EVs for up to 21 days in vitro and rat myocardial infarction models, and iCM-EVs are enriched with numerous heart-specific miRNAs, including miR-1 and miR133a, which can inhibit the rational hypertrophy of heart disease and reduce the size of myocardial infarction (Figure 2).

Ji et al. [57] encapsulated RGD hydrogels enriched in hypoxia-inducible factor-1α (HIF-1α)-overexpressing UCMSCs-derived engineered EVs (HIF-1α-EVs). The hydrogels inhibited the elevation of interleukin-6 (IL-6) and connexin 43 (conx43) in the region of myocardial infarction, which accelerated the recovery of cardiac function, reduced infarct size, and inhibited cardiomyocyte apoptosis.

Mehdi et al. [53] loaded two synthetic miRNA mimics, miR-126 and miR-146a, into the EVs secreted by adipose-derived mesenchymal stem cells (ADSCs) and assembled the EVs into injectable Ca-Alg hydrogels by in situ polymerization. miR-126 regulates the PI3K/AKT signaling pathway by upregulating vascular endothelial growth factor receptor 2 (VEGFR2), Protein kinase B (AKT), and the mechanistic target of rapamycin (mTOR), and inhibiting the expression of Sprouty-related EVH1 domain containing 1 (SPRED-1), thereby promoting cell migration and proliferation. miR-146a plays an anti-inflammatory role by inhibiting the expression of Interleukin-1 receptor-associated kinases (Irak-1) and tumor necrosis factor receptor-associated factor 6 (Traf6) genes, reducing pro-inflammatory cytokines. The composite hydrogel has been shown to reduce the size of myocardial infarction and fibrosis and promote angiogenesis in cardiac tissue.

### 4.2. Intervertebral Disc Degeneration

Intervertebral disc degeneration (IDD) is usually caused by the aging of intervertebral disc nucleus pulposus stem cells (NPSCs) [118]. In degenerated tissues, there is dysfunction in the surviving NPSCs [119], so it is difficult to achieve satisfactory therapeutic results by intervening in cell death or using stem cell therapy alone. Mesenchymal stem cell (MSCs)-based tissue engineering has the potential to treat cartilage defects [120], and MSCs secrete EVs rich in miRNAs associated with cartilage regeneration, demonstrating great therapeutic potentials due to their high stability, readily available, and abundant sources [121].

Shao et al. [58] isolated miR-3594-5p-enriched EVs from the medium of BMSCs, and combined them with RGD complex decellularized nucleus pulposus hydrogel (RGD-DNP) through the integrin expressed on the EVs membrane. The gel exhibited excellent cell-integration ability, and this study demonstrated for the first time that miR-3954-5p can effectively slow down cellular aging by targeting the 3′-UTR of the homeodomain-interacting protein kinase 2 (HIPK2) mRNA to reduce its expression.

Ye et al. [55] demonstrated, for the first time, the role of M2c-type macrophages (M2c) in the immune regulation of IDD tissues. After the lyophilization of the HA hydrogel, the EVs enriched with miR-124-3p released by M2c were loaded into a hydrogel through adsorption. The gel was implanted into the degenerative site of the tail vertebral disc in rats, and EVs were continuously released in vivo for 28 days. miR-124-3p down-regulated cartilage intermediate layer protein 2 (CILP-2) in NPCs, indirectly promoted the expression of extracellular matrix (ECM) protein (collagen type II (ColII) and aggrecan), and inhibited the expression of metalloproteinases (matrix metalloproteinase 13 (MMP13) and ADAM metallopeptidase with thrombospondin type 1 motif 5 (ADAMTS5)), while enhancing the conduction of the TGF-β pathway. The hydrogel improved the metabolism of the NPCs matrix, which was conducive to the long-term treatment of IDD.

**Figure 3:**
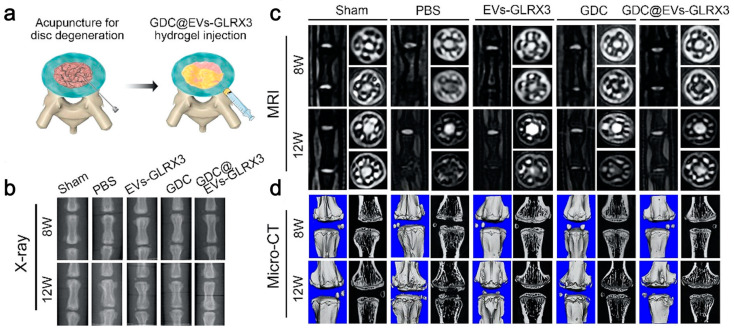
GLRX3-EVs-GDC slowed down disc degeneration. (**a**) The hydrogel was transplanted into the degenerative intervertebral disc; (**b**–**d**) representative images of X-ray, MRI, and Micro-CT of the discs at postoperative weeks 8 and 12 [82].

In addition, more and more research has shown that abnormal intracellular reactive oxygen species (ROS) levels are associated with NPSCs aging and are also a significant cause of IDD [122,123,124]. IDD can be slowed down by controlling the source ROS levels. Wang et al. [82] isolated the EVs with high expression of glutaredoxin3 (GLRX3) by treating BMSCs with hypoxia, and developed an injectable dopamine-grafted gelatin (GelDA) and aldehyde-functionalized chondroitin sulfate (ACS) composite hydrogel (GDC) for the delivery of EVs. GLRX3 inhibited the expression of P16INK4a, P21Cip1, and various cytosenescence-related factors including MMP13, interleukin-1β (IL-1β), and IL-6. The hydrogel has been shown to slow down mitochondrial damage in the rat IDD model to alleviate the aging of NPSCs, thereby slowing down IDD (Figure 3).

### 4.3. Osteoarthritis

Osteoarthritis (OA) is caused by changes in the composition or structure of any component of the joint, including cartilage fissures, chondrocyte apoptosis, etc. [125]. In addition, the articular cartilage cannot repair itself, and once damaged, it will continue to deteriorate [126]. OA is the leading cause of disability worldwide [127,128]. The role and mechanism of articular chondrocytes (ACs) and BMSCs in cartilage repair have been extensively studied [129,130,131], and their mechanisms of action typically involve EVs.

Cui et al. [61] isolated EVs secreted by subcutaneous adipose tissue-derived stem cells (ScASCs), and prepared injectable composite hydrogels of hydroxyacrylate polyethylene glycol diacrylate (HB-PEGDA) and mercaptoylated hyaluronic acid (SH-HA) (HB-PEGDA/SH-HA) in droplet-based microfluidic devices as an effective sustained-release carrier for EVs. miR-99a-3p is overexpressed in the EVs to inhibit the expression of ADAMTS4 and promote ECM repair. The composite hydrogel can be used for long-term treatment of OA.

Sun et al. [62] obtained EVs enriched with engineered miR-445 from transforming growth factor β3 (TGFβ3) pretreated BMSCs. The EVs were loaded into the gelatin–fibrinogen–HA–glycerol composite hydrogels by in situ polymerization, which were injected into rat knee joints. miR-445 regulates chondrogenesis and treats OA by targeting the SOX11/FOXO signaling pathway, reducing the expression of SRY-related HMG-box transcription factor 11 (SOX11), further enhancing the transcription of Forkhead box protein O1 (FOXO1), and upregulating the expression of SRY-related HMG-box transcription factor 9 (SOX9).

**Figure 4:**
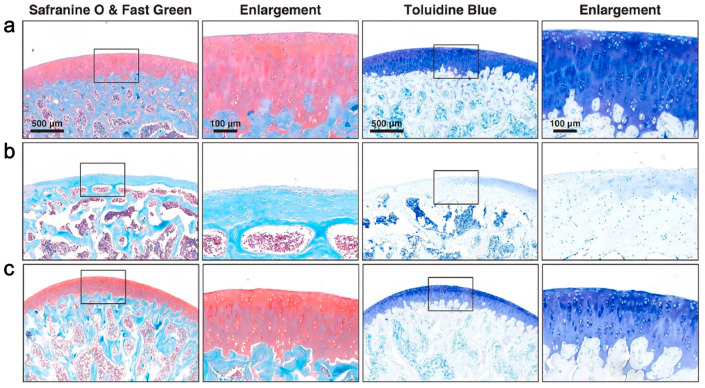
CircRNA3505-EVs-PLEL promoted the prevention of osteoarthritis. Histologic analysis with safranin O & fast green and toluidine blue staining for different groups: (**a**) normal; (**b**) osteoarthritis; (**c**) CircRNA3505-EVs-PLEL-treated (scale bar: 500 μm for low magnification and 100 μm for enlargement) [134].

Sleep is beneficial for cartilage repair [132], and circRNAs are involved in the pathogenesis of OA [133]. Guo et al. [134] successfully isolated circRNA3503-loaded EVs using melatonin (MT)-induced synovium mesenchymal stem cells (SMSCs). In this work, poly(D,_L_-lactide)-b-poly(ethylene glycol)-b-poly(D,_L_-lactide) (PDLLA-PEG-PDLLA) triblock copolymer gel (PLEL) was synthesized by in situ polymerization and used as the EV carrier for the first time. By inhibiting the expression of hsa-miR-181c-3p and hsa-let-7b-3p, circRNA3503 indirectly promotes the expression of peroxisome proliferator-activated receptor γ coactivator-1α (PGC-1α) and SOX9, thereby promoting chondrocyte renewal and treating OA (Figure 4).

### 4.4. Bone Deficiency

Bones could self-repair and regenerate after damage, and scar-free healing is achieved through the synergistic action of stem cells, progenitor cells, macrophages, etc. [135,136]. Despite this, the nonunion of fractures remains numerous and requires therapeutic interventions to promote bone repair and regeneration [137,138,139]. However, some growth factors such as recombinant human bone-forming proteins (rhBMPs) and platelet-derived growth factors (PDGFs) have been disappointing in their clinical and preclinical efficacy [140]. A growing body of research suggests that BMSCs play a crucial role in bone remodeling by secreting EVs [141,142].

Liu et al. [143] found that the EVs secreted by hypoxic pretreated BMSCs contained a large number of biglycans (Bgn). Liu and coworkers developed an injectable hydrogel composed of polyethylene glycol/polypeptide (PEG/PP) copolymer and mixed with EVs. The hydrogel could continuously release EVs for up to 3 weeks in a rat skull defect model. Bgn upregulates a variety of osteogenic properties-related genes including bone morphogenetic protein-2 (Bmp2), alkaline phosphatase (Alp), osteocalcin (Opn), Osteocalcin (Ocn), etc. Bgn also activates the PI3K/AKT signaling pathway and significantly promotes osteoblast differentiation.

**Figure 5:**
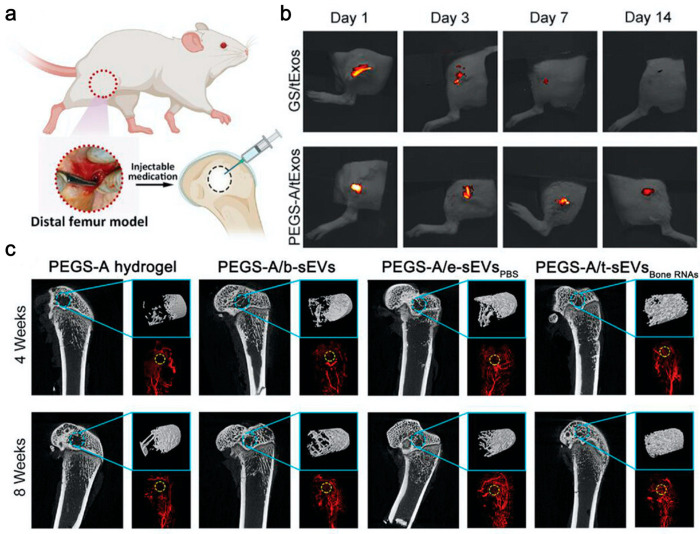
Bone RNAs-EVs-PEGSA accelerated bone regeneration and angiogenesis. (**a**) Injection treatment in rats with a critical-size femoral defect; (**b**) Fluorescence imaging of PKH26-labeled EVs delivered by gelatin sponge (GS) and PEGS-A in vivo; (**c**) Micro-CT images of new bone and vessel formation at weeks 4 and 8 [144].

Lee et al. [144] reported a cellular nanoelectroporation technique for the delivery of plasmids of Bmp2 and vascular endothelial growth factor A (VEGF-A) to human adipose-derived mesenchymal stem cells (hAdMSCs) and a large amount of mRNA was loaded in EVs secreted by plasmid-transfected hAdMSCs. By in situ polymerization, an EVs-loaded PEGylated poly (glycerol sebacate) acrylate (PEGS-A) injectable hydrogel was synthesized and the EVs were delivered locally in a controlled manner. In a rat model of a femoral defect, mRNA was efficiently expressed to achieve efficient angiogenesis and bone regeneration with less accumulation in other organs (Figure 5).

Based on the clinical evidence of traumatic brain injury (TBI) and accelerated bone healing, Bai et al. [145] proposed that EVs released by the damaged neurons were rich in miR-328a-3p and miR-150-5p, associated with bone formation, directly targeting the 3′UTR of forkhead box protein O4 (FOXO4) or calcineurin B-like protein (CBL) to promote bone formation. The skull defects were almost completely repaired after 3 months of injection of methacrylated glycol chitosan (MeGC) hydrogel carrying these EVs into a rat model of skull defects.

### 4.5. Diabetic Ulcer

About 20% of patients with diabetes have diabetic ulcers (DU) [146], the most common chronic wounds worldwide [147,148,149]. Incurable ulcers can seriously affect the patient’s quality of life, causing significant physical and psychological suffering. This refractory wound is caused by a range of therapeutic mechanisms, such as recurrent infections, delayed angiogenesis, impaired leukocyte function, and obstructed migration of keratinocytes, fibroblasts, and endothelial progenitor cells [150,151].

Zhou et al. [67] loaded EVs from BMSCs into a dopamine-modified GelMA hydrogel with tissue adhesion by in situ polymerization. The hydrogel increased the expression of IL-6, the cluster of differentiation 31 (CD31), and TGF-β in a diabetic rat skin wound model, significantly accelerating the wound closure rate and promoting healing.

Cui et al. [52] pretreated ADSCs with hypoxia, and separated and embedded their EVs on GelMA hydrogels by in situ polymerization. The EVs were rich in circ-Snhg11, which played a key role in wound healing, and by downregulating the expression of miR-144-3p, enhanced the expression of downstream nuclear factor erythroid 2-like 2 (NFE2L2) and HIF1α, enhancing the migration, proliferation, and revascularization of vascular endothelial cells (ECs) for the treatment of diabetic wounds.

**Figure 6:**
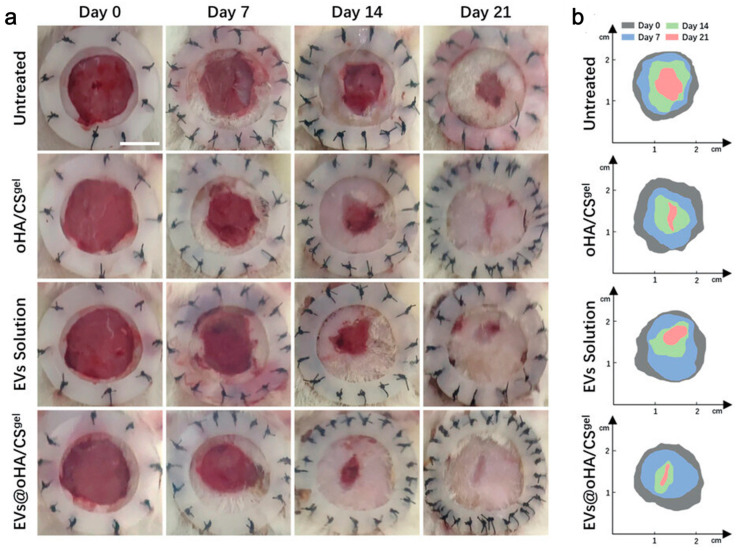
miR-7/21/221-EVs-oHA/CS accelerated wound healing of diabetic wounds. (**a**) Wound images on days 0, 7, 14, 21 after the first treatment; (**b**) schematic diagram of wound healing [54].

Based on the pathological features of abnormal hyperplasia of vascular endothelial cells in infantile hemangioma (IH) [152], Sha et al. [54] isolated CD133-positive Hemangioma stem cells (HemSCs) from IH and obtained the EVs from culture supernatants. They modified CS with hyaluronic oligosaccharides (oHA) to synthesize a thermosensitive hydrogel that served as a carrier for the EVs. In the EVs, the miR-7 family is highly expressed to promote angiogenesis, and miR-21 as well as miR-221 are highly expressed to promote endothelial cell proliferation. As a result, the composite hydrogel promoted wound healing (Figure 6).

### 4.6. Nerve Injury

Peripheral nerve injury (PNI), a disruption of bioelectrical communication between the spinal cord and the innervated body, can lead to chronic pain, muscle wasting, disability, and paralysis [153,154,155] and is a significant clinical challenge. Autologous nerve transplantation is currently a common strategy for the repair of PNI, but there are still shortcomings such as defects in donor tissue and cumbersome surgery [156]. Spinal cord injury (SCI) within the central nervous system is also one of the most devastating neurological diseases, with an estimated 180,000 new cases of SCI occurring each year worldwide [157] leading to long-term disability and complications such as neuroinflammation and oxidative damage [158,159]. Effective SCI remediation methods are still a huge challenge. Methods that utilize natural or synthetic materials as implantable nerve catheters or nerve scaffolds are promising in promoting nerve cell growth [160,161].

Han et al. [56] isolated NT-3 mRNA-rich EVs by transfecting ADSCs with lentivirus and encapsulated them in Ca-Alg hydrogels by in situ polymerization. The gel stably delivered NT-3 mRNA to targeted cellular SCs, effectively expressing neurotrophic factor 3 (NT-3) protein, and promoting peripheral nerve regeneration and functional recovery.

Qi et al. [162] used a commercially available injectable polyethylene glycol ether and polyethylene glycol triblock copolymer hydrogel (PLGA-PEG-PLGA) as a carrier for miR-138-5p modified UCMSCs EVs. The hydrogel was formed by dissolving PLGA-PEG-PLGA in a PBS solution containing EVs. In the SCI rat model, miR-138-5p decreased neuronal apoptosis by increasing the expression of nuclear factor erythrocyte 2-related factor 2 (Nrf2) protein thereby decreasing the expression of Kelch-like ECH-associated protein 1 (keap1), and it also played an anti-inflammatory role by decreasing the expression of NOD-like receptor thermal protein domain-associated protein 3 (NLRP3), thereby downregulating caspase-1. As a result, the EV-loaded hydrogel promoted the recovery of neurological function.

**Figure 7:**
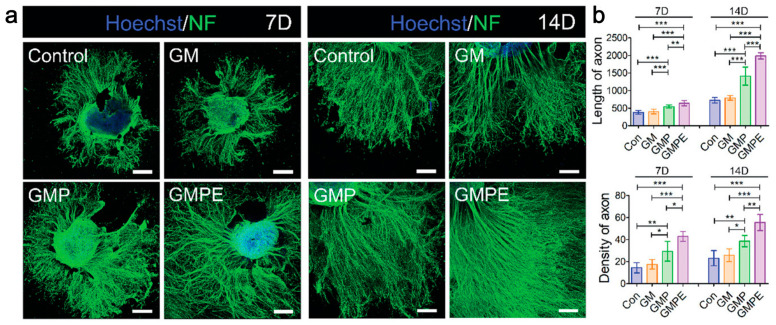
miRNA-EVs-GMP promoted axon growth and the formation of synaptic networks. (**a**) Immunofluorescence images of NF-positive axons in dorsal root ganglions (DRGs) cultured in hydrogels; (**b**) yhe density and length of axons were quantified. Statistical differences were determined using an ANOVA with Bonferroni’s multiple comparison test (* *p* < 0.05, ** *p* < 0.01, *** *p* < 0.001) [163].

Ning et al. [163] developed a double network gel of gelatin methacrylate and polypyrrole cross-linked tannins (GMP), loaded with EVs from BMSCs by adsorption. The EVs highly expressed miRNAs associated with axonal regeneration, myelination, and anti-inflammatory effects, and inhibited the expression of the proteins p-IKKα/β, p-IκBα, and p-P65, promoted the polarization of M2 microglia, increased the expression of axon-associated protein neurofilament (NF) and growth-associated protein-43 (GAP43), and promoted axon growth and the formation of synaptic networks (Figure 7).

### 4.7. Others

Malignancies are associated with high morbidity and mortality worldwide, and immunotherapy is an effective strategy for the treatment of malignant cancers [164], including adoptive cell immunotherapy, cancer vaccines, small molecule inhibitors, etc. [165]. Efforts have been underway for decades in the development of therapeutic cancer vaccines [166], and vaccines work primarily by activating systemic anti-tumor responses, whether hematologic or solid [167]. Yang et al. [168] developed a nano-clay GelMA hydrogel vaccine. The hydrogel was loaded with chemokine 21 (CCL21a) and tumor cell-derived EVs, which contained granulocyte-macrophage colony-stimulating factor (GM-CSF) mRNA and surface-modified sonosensitizer chlorin e6 (Ce6). CCL21a was responsible for recruiting tumor cells into the hydrogel, leading to EVs-induced immunogenic cell death (ICD) in cancer cells. Ce6-enhanced tumor cell phagocytosis of tumor cells in sonodynamic therapy. The hydrogel vaccine was shown to elicit effective anti-tumor immunity in colon and breast cancer mice.

Muscle wasting affects about 15% of people over the age of 65 [169] and is characterized by a decline in muscle function and mass [170]. Muscle wasting is usually caused by a loss of the self-renewal capacity of Schwann cells (SCs). Some miRNAs, such as miR-1 [171] and miR-133 [172], have been shown to play an important role in promoting muscle regeneration, while other miRNAs are pathogenic to muscle wasting, such as miR-29b [173] and miR-628 [174]. Xu et al. [175] showed that miR-467a-3p and miR-874-5p inhibited the differentiation of SCs and the formation of muscle tissue, respectively. They overexpressed TSG101 to target SCs on the BMSCs-EVs surface, and transported antagomiR-467a-3p and antagomiR-874-5p, respectively, to construct two engineered EVs. Sodium alginate (SA) and Pluronic F-127 (PF127) were used to form a layered injectable hydrogel. With the EV loaded in the hydrogel, the EVs containing two antagomirs were released in vivo for the treatment of muscle atrophy (MA).

Postoperative pericardial adhesion (PPA) is a fibrous connection between the epicardium and the thoracic cavity [176,177]. PPA reduces the surgical field of view and prolongs the operative time during cardiac surgical reoperation [178]. PPA also limits left ventricular diastolic filling, leading to myocardial infarction and, in severe cases, sudden death [179]. The molecular mechanism of PPA has not been fully elucidated, and effective methods to prevent PPA are urgently needed. Wu et al. [180] encapsulated the EVs from iCMs in a hyaluronic acid–g-(2-aminoethyl methacrylate hydrochloride–dopamine) (HAD) hydrogel by in situ polymerization under 365 nm of UV irradiation (7 mW/cm^2^) for 10 s. The EVs-loaded hydrogel inhibited Nrf2, alleviating oxidative stress in primary cardiomyocytes, and downregulated interleukin-1β (IL-1β), tumor necrosis factor-α (TNF-α), and IL-6, exerting anti-inflammatory effects. In addition, HAD hydrogel acted as a polyanion trap to prevent PPA by neutralizing MSR-1 scavenger receptors and inhibiting the recruitment of GATA6+ macrophages to adhesion sites [181].

Acute kidney injury (AKI) occurs in approximately 32% of patients admitted to the intensive care unit (ICU) and 24% of patients undergoing cardiac surgery [182], and there is currently no effective treatment to prevent AKI, so there is an urgent need for new renal-protective therapies [183]. Chen et al. [59] developed an RGD hydrogel as a scaffold for the EVs of human placenta-derived mesenchymal stem cells (hP-MSCs) by in situ polymerization. miR-let-7a-5p in EVs was key to reducing renal apoptosis and enhance autophagy in AKI by targeting inhibition of caspase-3 (CASP3) mRNA and ras-related GTP binding D (RragD) mRNA, thereby exerting renal protective effects.

With the improvement of life quality, EVs have also attracted increasing interest from the beauty industry. Dermatologists and cosmetologists are committed to skin renewal and repair, and anti-wrinkle is one of the top research priorities [184,185]. HA-based hydrogels are used to develop new fillers that are highly effective in repairing the damaged dermal microenvironment [186]. Park et al. [187] extracted EVs from ADSCs and conjugated them to HA hydrogels by adsorption. miRNA-let-7b-5p and miR-24-3p in the EVs induced overexpression of CD301b in macrophages and promoted the proliferation of fibroblasts, demonstrating the anti-aging potential of the EV-loaded HA hydrogel.

## 5. Implications, Limitations and Future

In recent years, EVs have attracted a lot of attention for CFTs [188], which can be isolated from human cells, body fluids, milk, bacteria, etc. [189,190,191]. Hydrogels have a good biocompatibility, excellent biodegradability, and a high loading capacity, and can be designed as tissue patches or intravenous and subcutaneous injectable agents [192,193]. Not surprisingly, hydrogels have become an excellent EVs delivery platform, which can control the release of EVs through swelling [194] or degradation [195] and solves the problems of short intravenous half-life of EVs, rapid immune clearance, and liver accumulation [196]. Composite hydrogels containing EVs derived from different cells can be used as an effective CFT in many diseases such as myocardial infarction and osteoarthritis (Table 1).

However, there are still significant challenges before the hydrogels loaded with EVs can be translated into clinical practice. The first group of challenges are associated with the hydrogels. The stability and EV-release characteristics of hydrogels are affected by the hydrogels’ synthesis conditions and in vivo tissue temperature [197]. The hydrogels based on natural biomaterials often have low mechanical strength [198,199]. The residual monomers or crosslinkers in the hydrogels may present biosafety issues [200]. Therefore, new hydrogel materials need to be designed and synthesized. In addition, the large-scale production of some hydrogels is needed [201]. The second group of challenges are associated with EVs. The purity of the EVs, commonly isolated by ultracentrifugation, needs to be improved [30,202]. The heterogeneity in the source of EVs will lead to different therapeutic effects [203]. EVs are stored at –80 °C, and repeated freeze–thaw processes may lead to the deterioration of bioactive molecules [204]. Finally, in the preparation of EVs-loaded hydrogels by in situ polymerization method, in order not to affect the activity of EVs, the optimal reaction conditions of the in situ polymerization still need to be explored [205].

As the field of hydrogel materials continues to advance, challenges associated with EVs-loaded hydrogels are progressively being addressed [206]. For example, by selecting suitable hydrogel materials that cater to specific release characteristics, the controlled release of EVs can be finely tuned. This includes options for short-term and long-term release, as well as tailored approaches like continuous or pulsatile release modes, thereby expanding the versatility and applicability of these systems in therapeutic scenarios [74]. Hydrogels with higher ester bonds such as 8-Arm-Poly(ethylene glycol)-mono(2-acryloyloxyethyl) succinate (8-Arm-PEG-MAES) exhibit faster swelling and degradation [207], resulting in a faster release of EVs. Additionally, by modifying the ratio of hydrophobic networks, the hydrophilicity of the hydrogel network can be adjusted, enabling control over the swelling rate, and thereby realizing a time-regulated release of EVs [208]. The passively controlled release of EVs can be achieved through stimuli-responsive hydrogels, such as isoguanosine-phenylboronic acid-guanosine (isoGPBG) pH-responsive hydrogels that rely on Schiff base bonds [209] and hydrazine or aldehyde-modified hyaluronic acid (HA) enzyme-sensitive hydrogels containing enzyme-sensitive peptides [210]. Furthermore, the separation and purification of EVs can be efficiently accomplished using microfluidic technology, which allows for the effective segregation of EVs from small sample volumes and their classification and extraction based on size [211,212,213]. In the preparation of EVs-loaded hydrogels, the adsorption in pre-formed hydrogels is more advantageous over in situ polymerization, because it is much easier to remove the residual monomers, cross-linked agents, initiators, toxic metal ions and other impurities from the pre-formed hydrogels [163,214,215,216].

In addition to accurate drug release and targeted treatment in disease treatment, EVs-loaded hydrogels are also useful in the fundamental research in life science, such as intercellular interactions, signal transmission, molecular communication, etc. [217,218].

For future preclinical studies, two important directions include identifying the optimal cell source of the EVs and developing new composite hydrogel materials. It is also crucial to evaluate the benefit–risk ratio of each EVs-loaded hydrogel, including production cost, potential contamination in the production, long-term safety and efficacy, etc. Overall, EVs-loaded hydrogels hold tremendous promise in cell-free therapy strategies.

## Figures and Tables

**Table 1:** Applications of EV-loaded hydrogel in the treatment of diseases.

Origins of EVs	Preprocessing Method	Biological Molecules	Hydrogels	Target Disease	Therapeutic Effect	Reference
iCMs		miR-1, miR133a	collagen	MI	Inhibit the rational hypertrophy of heart disease and reduce the size of myocardial infarction	[117]
		N/A	HAD	PPA	Prevent PPA	[180]
UCMSCs	Cells transduced with an HIF-1α-overexpressing lentivirus	HIF-1α	RGD	MI	Accelerate the recovery of cardiac function, reduce infarct size, and inhibit cardiomyocyte apoptosis	[57]
	EVs loaded with miRNA mimics	miR-138-5p	PLGA-PEG-PLGA	SCI	Promote the recovery of neurological function	[162]
ADSCs	EVs loaded with miRNA mimics	miR-126, miR-146a	Ca-Alg	MI	Reduce the size of myocardial infarction and fibrosis and promote angiogenesis in cardiac tissue	[53]
		miR-99b-3p	HB-PEGDA/SH-HA	OA	Accelerate cartilage repair	[61]
	Cells transduced with plasmids of Bmp2 and VEGF-A	Bmp2 mRNA and VEGF-A mRNA	PEGS-A	BD ^1^	Achieve efficient angiogenesis and bone regeneration	[144]
	Cells treated with hypoxia	circ-Snhg11	GelMA	DU	Promote wound healing in diabetes	[52]
	Cells transduced with NT-3 plasmid	NT-3 mRNA	Ca-Alg	PNI	Promote peripheral nerve regeneration and functional recovery	[56]
		miRNA-let-7b-5p and miR-24-3p	HA	wrinkles	Reduce wrinkles	[187]
BMSCs		miR-3594-5p	RGD-DNP	IDD	Slow down IDD	[58]
	Cells treated with hypoxia	GLRX3	GDC	IDD	Slow down IDD	[82]
	Cells treated with TGFβ3	miR-445	GFHG ^2^	OA	Accelerate cartilage repair	[62]
	Cells treated with hypoxia	Bgn	PEG-PP	BD	Accelerate bone regeneration	[143]
		N/A	GelMA	DU	Accelerate the wound closure rate and promote healing.	[67]
		miRNAs	GM-PPy-TA	SCI	Promote axon growth and the formation of synaptic networks	[163]
	EVs loaded with antagomirs	antagomiR-467a-3p and antagomiR-874-5p	Alg-PF127	MA	Promote the formation of muscle tissue	[175]
BMDMs	Cells induced M2c Polarization by IL-10	miR-124-3p	HA	IDD	Slow down IDD	[55]
SMSCs	Cells treated with MT	circRNA3503	PDLLA-PEG-PDLLA	OA	Promote cartilage repair	[134]
TBI		miR-328a-3p and miR-150-5p	MeGC	BD	Accelerate bone re-generation	[145]
HemSCs		miR-7, miR-21 and miR-221	oHA- CS	DU	Promote wound healing	[54]
tumor cells	Cells transfected with pcDNA3.1(-)-GM-CSF plasmid	GM-CSF mRNA	Nano clay-GelMA	Malignancies	Achieve effective anti-tumor immunity	[168]
hP-MSCs		miR-let-7a-5p	RGD	AKI	Prevent AKI	[59]

^1^ Bone deficiency. ^2^ Gelatin-fibrinogen-hyaluronic acid-glycerol composite hydrogel.

## References

[1] Miyahara Y., Nagaya N., Kataoka M., Yanagawa B., Tanaka K., Hao H., Ishino K., Ishida H., Shimizu T., Kangawa K. (2006). Monolayered mesenchymal stem cells repair scarred myocardium after myocardial infarction. Nat. Med..

[2] Mangi A.A., Noiseux N., Kong D., He H., Rezvani M., Ingwall J.S., Dzau V.J. (2003). Mesenchymal stem cells modified with Akt prevent remodeling and restore performance of infarcted hearts. Nat. Med..

[3] Dodson B.P., Levine A.D. (2015). Challenges in the translation and commercialization of cell therapies. BMC Biotechnol..

[4] Fox I.J., Daley G.Q., Goldman S.A., Huard J., Kamp T.J., Trucco M. (2014). Use of differentiated pluripotent stem cells in replacement therapy for treating disease. Science.

[5] Malda J., Boere J., van de Lest C.H.A., van Weeren P.R., Wauben M.H.M. (2016). Extracellular vesicles—New tool for joint repair and regeneration. Nat. Rev. Rheumatol..

[6] Re F., Gabusi E., Manferdini C., Russo D., Lisignoli G. (2021). Bone regeneration improves with mesenchymal stem cell derived extracellular vesicles (EVs) combined with scaffolds: A systematic review. Biology.

[7] Duran P., Boscolo Sesillo F., Cook M., Burnett L., Menefee S.A., Do E., French S., Zazueta-Damian G., Dzieciatkowska M., Saviola A.J. (2023). Proregenerative extracellular matrix hydrogel mitigates pathological alterations of pelvic skeletal muscles after birth injury. Sci. Transl. Med..

[8] Liu Y., Guo R., Wu T., Lyu Y., Xiao M., He B., Fan G., Yang J., Liu W. (2021). One zwitterionic injectable hydrogel with ion conductivity enables efficient restoration of cardiac function after myocardial infarction. Chem. Eng. J..

[9] Shi M., Dong R., Hu J., Guo B. (2023). Conductive self-healing biodegradable hydrogel based on hyaluronic acid-grafted-polyaniline as cell recruitment niches and cell delivery carrier for myogenic differentiation and skeletal muscle regeneration. Chem. Eng. J..

[10] Wang L., Liu Y., Ye G., He Y., Li B., Guan Y., Gong B., Mequanint K., Xing M.M.Q., Qiu X. (2021). Injectable and conductive cardiac patches repair infarcted myocardium in rats and minipigs. Nat. Biomed. Eng..

[11] Wang L., Zhang X., He Y., Wang Y., Zhong W., Mequanint K., Qiu X., Xing M. (2019). Ultralight conductive and elastic aerogel for skeletal muscle atrophy regeneration. Adv. Funct. Mater..

[12] Baglio S.R., Pegtel D., Baldini N. (2012). Mesenchymal stem cell secreted vesicles provide novel opportunities in (stem) cell-free therapy. Front. Physiol..

[13] Zhang X., Zhang Y., Zhang R., Jiang X., Midgley A.C., Liu Q., Kang H., Wu J., Khalique A., Qian M. (2022). Biomimetic design of artificial hybrid nanocells for boosted vascular regeneration in ischemic tissues. Adv. Mater..

[14] Kahlert C., Melo S.A., Protopopov A., Tang J., Seth S., Koch M., Zhang J., Weitz J., Chin L., Futreal A. (2014). Identification of double-stranded genomic DNA spanning all chromosomes with mutated KRAS and p53 DNA in the serum exosomes of patients with pancreatic cancer. J. Biol. Chem..

[15] Xiong Y., Mahmood A., Chopp M. (2024). Mesenchymal stem cell-derived extracellular vesicles as a cell-free therapy for traumatic brain injury via neuroprotection and neurorestoration. Neural Regen. Res..

[16] Cocozza F., Grisard E., Martin-Jaular L., Mathieu M., Théry C. (2020). SnapShot: Extracellular vesicles. Cell.

[17] Kalluri R., McAndrews K.M. (2023). The role of extracellular vesicles in cancer. Cell.

[18] Bertolino G.M., Maumus M., Jorgensen C., Noël D. (2023). Therapeutic potential in rheumatic diseases of extracellular vesicles derived from mesenchymal stromal cells. Nat. Rev. Rheumatol..

[19] O’Reilly D., Egan K., Burke O., Griffiths A., Neary E., Blanco A., Szklanna P., Maguire P., McCallion N., Ni Ainle F. (2018). The population of circulating extracellular vesicles dramatically alters after very premature delivery—A previously unrecognised postnatal adaptation process?. Blood.

[20] Kalluri R., LeBleu V.S. (2020). The biology, function, and biomedical applications of exosomes. Science.

[21] Borgovan T., Nwizu C.C., Goldberg L.R., Dooner M.S., Wen S., Deltatto M., Crawford L., Quesenberry P.J. (2018). Extracellular vesicles (EVs) shape the leukemic microenvironment. Blood.

[22] Pakravan K., Babashah S., Sadeghizadeh M., Mowla S.J., Mossahebi-Mohammadi M., Ataei F., Dana N., Javan M. (2017). MicroRNA-100 shuttled by mesenchymal stem cell-derived exosomes suppresses in vitro angiogenesis through modulating the mTOR/HIF-1α/VEGF signaling axis in breast cancer cells. Cell. Oncol..

[23] Nam G.-H., Choi Y., Kim G.B., Kim S., Kim S.A., Kim I.-S. (2020). Emerging prospects of exosomes for cancer treatment: From conventional therapy to immunotherapy. Adv. Mater..

[24] Takahashi Y., Nishikawa M., Shinotsuka H., Matsui Y., Ohara S., Imai T., Takakura Y. (2013). Visualization and in vivo tracking of the exosomes of murine melanoma B16-BL6 cells in mice after intravenous injection. J. Biotechnol..

[25] Imai T., Takahashi Y., Nishikawa M., Kato K., Morishita M., Yamashita T., Matsumoto A., Charoenviriyakul C., Takakura Y. (2015). Macrophage-dependent clearance of systemically administered B16BL6-derived exosomes from the blood circulation in mice. J. Extracell. Vesicles.

[26] Zhang G., Huang X., Xiu H., Sun Y., Chen J., Cheng G., Song Z., Peng Y., Shen Y., Wang J. (2020). Extracellular vesicles: Natural liver-accumulating drug delivery vehicles for the treatment of liver diseases. J. Extracell. Vesicles.

[27] Lin J., Wang Z., Huang J., Tang S., Saiding Q., Zhu Q., Cui W. (2021). Microenvironment-protected exosome-hydrogel for facilitating endometrial regeneration, fertility restoration, and live birth of offspring. Small.

[28] Wu R., Fan X., Wang Y., Shen M., Zheng Y., Zhao S., Yang L. (2022). Mesenchymal stem cell-derived extracellular vesicles in liver immunity and therapy. Front. Immunol..

[29] Herrmann I.K., Wood M.J.A., Fuhrmann G. (2021). Extracellular vesicles as a next-generation drug delivery platform. Nat. Nanotechnol..

[30] Kordelas L., Rebmann V., Ludwig A.K., Radtke S., Ruesing J., Doeppner T.R., Epple M., Horn P.A., Beelen D.W., Giebel B. (2014). MSC-derived exosomes: A novel tool to treat therapy-refractory graft-versus-host disease. Leukemia.

[31] Dempsey L.A. (2023). Modulating bone marrow niches. Nat. Immunol..

[32] Morganstein D.L., Wu P., Mane M.R., Fisk N.M., White R., Parker M.G. (2010). Human fetal mesenchymal stem cells differentiate into brown and white adipocytes: A role for ERRα in human UCP1 expression. Cell Res..

[33] Zhang Y., Xie Y., Hao Z., Zhou P., Wang P., Fang S., Li L., Xu S., Xia Y. (2021). Umbilical mesenchymal stem cell-derived exosome-encapsulated hydrogels accelerate bone repair by enhancing angiogenesis. ACS Appl. Mater. Interfaces.

[34] Guan P., Liu C., Xie D., Mao S., Ji Y., Lin Y., Chen Z., Wang Q., Fan L., Sun Y. (2022). Exosome-loaded extracellular matrix-mimic hydrogel with anti-inflammatory property Facilitates/promotes growth plate injury repair. Bioact. Mater..

[35] Sun Y., Sun Y., Chen S., Yu Y., Ma Y., Sun F. (2023). Hypoxic preconditioned MSCs-derived small extracellular vesicles for photoreceptor protection in retinal degeneration. J. Nanobiotechnol..

[36] Hu X., Wu R., Shehadeh L.A., Zhou Q., Jiang C., Huang X., Zhang L., Gao F., Liu X., Yu H. (2014). Severe hypoxia exerts parallel and cell-specific regulation of gene expression and alternative splicing in human mesenchymal stem cells. BMC Genom..

[37] Feng R., Mayuranathan T., Huang P., Doerfler P.A., Li Y., Yao Y., Zhang J., Palmer L.E., Mayberry K., Christakopoulos G.E. (2022). Activation of γ-globin expression by hypoxia-inducible factor 1α. Nature.

[38] Thienpont B., Steinbacher J., Zhao H., D’Anna F., Kuchnio A., Ploumakis A., Ghesquière B., Van Dyck L., Boeckx B., Schoonjans L. (2016). Tumour hypoxia causes DNA hypermethylation by reducing TET activity. Nature.

[39] Ito Y., Matsuzaki T., Ayabe F., Mokuda S., Kurimoto R., Matsushima T., Tabata Y., Inotsume M., Tsutsumi H., Liu L. (2021). Both microRNA-455-5p and -3p repress hypoxia-inducible factor-2α expression and coordinately regulate cartilage homeostasis. Nat. Commun..

[40] Zhu L.-P., Tian T., Wang J.-Y., He J.-N., Chen T., Pan M., Xu L., Zhang H.-X., Qiu X.-T., Li C.-C. (2018). Hypoxia-elicited mesenchymal stem cell-derived exosomes facilitates cardiac repair through miR-125b-mediated prevention of cell death in myocardial infarction. Theranostics.

[41] Shen K., Duan A., Cheng J., Yuan T., Zhou J., Song H., Chen Z., Wan B., Liu J., Zhang X. (2022). Exosomes derived from hypoxia preconditioned mesenchymal stem cells laden in a silk hydrogel promote cartilage regeneration via the miR-205–5p/PTEN/AKT pathway. Acta Biomater..

[42] Rufino-Ramos D., Leandro K., Perdigão P.R.L., O’Brien K., Pinto M.M., Santana M.M., van Solinge T.S., Mahjoum S., Breakefield X.O., Breyne K. (2023). Extracellular communication between brain cells through functional transfer of Cre mRNA mediated by extracellular vesicles. Mol. Ther..

[43] Shen Y., Xu G., Huang H., Wang K., Wang H., Lang M., Gao H., Zhao S. (2021). Sequential release of small extracellular vesicles from bilayered thiolated alginate/polyethylene glycol diacrylate hydrogels for scarless wound healing. ACS Nano.

[44] Zhai X., Chen K., Yang H., Li B., Zhou T., Wang H., Zhou H., Chen S., Zhou X., Wei X. (2021). Extracellular vesicles derived from CD73 modified human umbilical cord mesenchymal stem cells ameliorate inflammation after spinal cord injury. J. Nanobiotechnol..

[45] Nguyen V.-N., Dao T.N.T., Cho M., Jeong H., Nguyen-Le M.-T., Shin Y., Yoon J. (2023). Recent advances in extracellular vesicle-based organic nanotherapeutic drugs for precision cancer therapy. Coord. Chem. Rev..

[46] Verweij F.J., Balaj L., Boulanger C.M., Carter D.R.F., Compeer E.B., D’Angelo G., El Andaloussi S., Goetz J.G., Gross J.C., Hyenne V. (2021). The power of imaging to understand extracellular vesicle biology in vivo. Nat. Methods.

[47] Ding Y.-N., Ding H.-Y., Li H., Yang R., Huang J.-Y., Chen H., Wang L.-H., Wang Y.-J., Hu C.-M., An Y.-L. (2023). Photosensitive small extracellular vesicles regulate the immune microenvironment of triple negative breast cancer. Acta Biomater..

[48] Chen C., Sun M., Wang J., Su L., Lin J., Yan X. (2021). Active cargo loading into extracellular vesicles: Highlights the heterogeneous encapsulation behaviour. J. Extracell. Vesicles.

[49] Cao Z., Li P., Li Y., Zhang M., Hao M., Li W., Mao X., Mo L., Yang C., Ding X. (2023). Encapsulation of nano-bortezomib in apoptotic stem cell-derived vesicles for the treatment of multiple myeloma. Small.

[50] Syn N.L., Wang L., Chow E.K.-H., Lim C.T., Goh B.-C. (2017). Exosomes in cancer nanomedicine and immunotherapy: Prospects and challenges. Trends Biotechnol..

[51] Yerneni S.S., Lathwal S., Cuthbert J., Kapil K., Szczepaniak G., Jeong J., Das S.R., Campbell P.G., Matyjaszewski K. (2022). Controlled release of exosomes using atom transfer radical polymerization-based hydrogels. Biomacromolecules.

[52] Hu N., Cai Z., Jiang X., Wang C., Tang T., Xu T., Chen H., Li X., Du X., Cui W. (2023). Hypoxia-pretreated ADSC-derived exosome-embedded hydrogels promote angiogenesis and accelerate diabetic wound healing. Acta Biomater..

[53] Shafei S., Khanmohammadi M., Ghanbari H., Nooshabadi V.T., Tafti S.H.A., Rabbani S., Kasaiyan M., Basiri M., Tavoosidana G. (2022). Effectiveness of exosome mediated miR-126 and miR-146a delivery on cardiac tissue regeneration. Cell Tissue Res..

[54] Lu E., Yang X., Wang T., Huang X., Chen Y., Wang R., Luo K., Zhang Z., Lin X., Sha X. (2023). Biomimetic thermo-sensitive hydrogel encapsulating hemangiomas stem cell derived extracellular vesicles promotes microcirculation reconstruction in diabetic wounds. Adv. Funct. Mater..

[55] Liu Y., Xue M., Han Y., Li Y., Xiao B., Wang W., Yu J., Ye X. (2023). Exosomes from M2c macrophages alleviate intervertebral disc degeneration by promoting synthesis of the extracellular matrix via MiR-124/CILP/TGF-β. Bioeng. Transl. Med..

[56] Yang Z., Yang Y., Xu Y., Jiang W., Shao Y., Xing J., Chen Y., Han Y. (2021). Biomimetic nerve guidance conduit containing engineered exosomes of adipose-derived stem cells promotes peripheral nerve regeneration. Stem Cell Res. Ther..

[57] Wang Q., Zhang L., Sun Z., Chi B., Zou A., Mao L., Xiong X., Jiang J., Sun L., Zhu W. (2021). HIF-1α overexpression in mesenchymal stem cell-derived exosome-encapsulated arginine-glycine-aspartate (RGD) hydrogels boost therapeutic efficacy of cardiac repair after myocardial infarction. Mater. Today Bio.

[58] Peng Y., Chen X., Liu S., Wu W., Shu H., Tian S., Xiao Y., Li K., Wang B., Lin H. (2023). Extracellular vesicle-conjugated functional matrix hydrogels prevent senescence by exosomal miR-3594-5p-targeted HIPK2/p53 pathway for disc regeneration. Small.

[59] Zhang C., Shang Y., Chen X., Midgley A.C., Wang Z., Zhu D., Wu J., Chen P., Wu L., Wang X. (2020). Supramolecular nanofibers containing arginine-glycine-aspartate (rgd) peptides boost therapeutic efficacy of extracellular vesicles in kidney repair. ACS Nano.

[60] Xu Y., Qiu Y., Lin Q., Huang C., Li J., Chen L., Xue Z., Wu Q., Wang Y. (2022). miR-126-3p-loaded small extracellular vesicles secreted by urine-derived stem cells released from a phototriggered imine crosslink hydrogel could enhance vaginal epithelization after vaginoplasty. Stem Cell Res. Ther..

[61] Yin Z., Qin C., Pan S., Shi C., Wu G., Feng Y., Zhang J., Yu Z., Liang B., Gui J. (2023). Injectable hyperbranched PEG crosslinked hyaluronan hydrogel microparticles containing mir-99a-3p modified subcutaneous ADSCs-derived exosomes was beneficial for long-term treatment of osteoarthritis. Mater. Today Bio.

[62] Sun Y., Zhao J., Wu Q., Zhang Y., You Y., Jiang W., Dai K. (2022). Chondrogenic primed extracellular vesicles activate miR-455/SOX11/FOXO axis for cartilage regeneration and osteoarthritis treatment. npj Regen. Med..

[63] Li M., Ke Q.-F., Tao S.-C., Guo S.-C., Rui B.-Y., Guo Y.-P. (2016). Fabrication of hydroxyapatite/chitosan composite hydrogels loaded with exosomes derived from miR-126-3p overexpressed synovial mesenchymal stem cells for diabetic chronic wound healing. J. Mater. Chem. B.

[64] Tang Q., Lu B., He J., Chen X., Fu Q., Han H., Luo C., Yin H., Qin Z., Lyu D. (2022). Exosomes-loaded thermosensitive hydrogels for corneal epithelium and stroma regeneration. Biomaterials.

[65] Kuang H., Ma J., Chi X., Fu Q., Zhu Q., Cao W., Zhang P., Xie X. (2023). Integrated osteoinductive factors─exosome@microrna-26a hydrogel enhances bone regeneration. ACS Appl. Mater. Interfaces.

[66] Wu D., Qin H., Wang Z., Yu M., Liu Z., Peng H., Liang L., Zhang C., Wei X. (2022). Bone mesenchymal stem cell-derived sev-encapsulated thermosensitive hydrogels accelerate osteogenesis and angiogenesis by release of exosomal mir-21. Front. Bioeng. Biotechnol..

[67] Wang Y., Song P., Wu L., Su Z., Gui X., Gao C., Zhao H., Wang Y., Li Z., Cen Y. (2023). In situ photo-crosslinked adhesive hydrogel loaded with mesenchymal stem cell-derived extracellular vesicles promotes diabetic wound healing. J. Mater. Chem. B.

[68] Hu H., Dong L., Bu Z., Shen Y., Luo J., Zhang H., Zhao S., Lv F., Liu Z. (2020). miR-23a-3p-abundant small extracellular vesicles released from Gelma/nanoclay hydrogel for cartilage regeneration. J. Extracell. Vesicles.

[69] Taylor D.L., Panhuis M.I.N. (2016). Self-healing hydrogels. Adv. Mater..

[70] Zhang Z., He C., Chen X. (2024). Designing hydrogels for immunomodulation in cancer therapy and regenerative medicine. Adv. Mater..

[71] Li Y., Chen R., Zhou B., Dong Y., Liu D. (2023). Rational design of dna hydrogels based on molecular dynamics of polymers. Adv. Mater..

[72] Shan B.-H., Wu F.-G. (2023). Hydrogel-based growth factor delivery platforms: Strategies and recent advances. Adv. Mater..

[73] Erfani A., Diaz A.E., Doyle P.S. (2023). Hydrogel-enabled, local administration and combinatorial delivery of immunotherapies for cancer treatment. Mater. Today.

[74] Zhong R., Talebian S., Mendes B.B., Wallace G., Langer R., Conde J., Shi J. (2023). Hydrogels for RNA delivery. Nat. Mater..

[75] Allen M.E., Hindley J.W., Baxani D.K., Ces O., Elani Y. (2022). Hydrogels as functional components in artificial cell systems. Nat. Rev. Chem..

[76] Zhu J.-Q., Wu H., Li Z.-L., Xu X.-F., Xing H., Wang M.-D., Jia H.-D., Liang L., Li C., Sun L.-Y. (2022). Responsive hydrogels based on triggered click reactions for liver cancer. Adv. Mater..

[77] Yin Y., Gu Q., Liu X., Liu F., McClements D.J. (2023). Double network hydrogels: Design, fabrication, and application in biomedicines and foods. Adv. Colloid Interface Sci..

[78] Khalesi H., Lu W., Nishinari K., Fang Y. (2020). New insights into food hydrogels with reinforced mechanical properties: A review on innovative strategies. Adv. Colloid Interface Sci..

[79] Yang J., Li K., Tang C., Liu Z., Fan J., Qin G., Cui W., Zhu L., Chen Q. (2022). Recent progress in double network elastomers: One plus one is greater than two. Adv. Funct. Mater..

[80] Li K., Chen Y., Li A., Tan C., Liu X. (2019). Exosomes play roles in sequential processes of tumor metastasis. Int. J. Cancer.

[81] Wang H., Cui J., Zheng Z., Shi Q., Sun T., Liu X., Huang Q., Fukuda T. (2017). Assembly of RGD-modified hydrogel micromodules into permeable three-dimensional hollow microtissues mimicking in vivo tissue structures. ACS Appl. Mater. Interfaces.

[82] Liu C., Fan L., Guan M., Zheng Q., Jin J., Kang X., Gao Z., Deng X., Shen Y., Chu G. (2023). A redox homeostasis modulatory hydrogel with GLRX3^+^ extracellular vesicles attenuates disc degeneration by suppressing nucleus pulposus cell senescence. ACS Nano.

[83] Dixson A.C., Dawson T.R., Di Vizio D., Weaver A.M. (2023). Context-specific regulation of extracellular vesicle biogenesis and cargo selection. Nat. Rev. Mol. Cell Biol..

[84] Staufer O., Hernandez Bücher J.E., Fichtler J., Schröter M., Platzman I., Spatz J.P. (2022). Vesicle induced receptor sequestration: Mechanisms behind extracellular vesicle-based protein signaling. Adv. Sci..

[85] Yang Y., Hong Y., Cho E., Kim G.B., Kim I.-S. (2018). Extracellular vesicles as a platform for membrane-associated therapeutic protein delivery. J. Extracell. Vesicles.

[86] Sterzenbach U., Putz U., Low L.-H., Silke J., Tan S.-S., Howitt J. (2017). Engineered exosomes as vehicles for biologically active proteins. Mol. Ther..

[87] Polack F.P., Thomas S.J., Kitchin N., Absalon J., Gurtman A., Lockhart S., Perez J.L., Pérez Marc G., Moreira E.D., Zerbini C. (2020). Safety and Efficacy of the BNT162b2 mRNA COVID-19 Vaccine. N. Engl. J. Med..

[88] Morris V.K., Kopetz S. (2022). Don’t blame the messenger: Lessons learned for cancer mRNA vaccines during the COVID-19 pandemic. Nat. Rev. Cancer.

[89] Tang Z., Yu F., Hsu J.C., Shi J., Cai W. (2023). Soybean oil-derived lipids for efficient mrna delivery. Adv. Mater..

[90] Zhu Y., Ma J., Shen R., Lin J., Li S., Lu X., Stelzel J.L., Kong J., Cheng L., Vuong I. (2023). Screening for lipid nanoparticles that modulate the immune activity of helper T cells towards enhanced antitumour activity. Nat. Biomed. Eng..

[91] Yang Z., Shi J., Xie J., Wang Y., Sun J., Liu T., Zhao Y., Zhao X., Wang X., Ma Y. (2020). Large-scale generation of functional mRNA-encapsulating exosomes via cellular nanoporation. Nat. Biomed. Eng..

[92] Stoorvogel W. (2012). Functional transfer of microRNA by exosomes. Blood.

[93] Sun R., Zhang P.-P., Weng X.-Q., Gao X.-D., Huang C.-X., Wang L., Hu X.-X., Xu P.-P., Cheng L., Jiang L. (2022). Therapeutic targeting miR130b counteracts diffuse large B-cell lymphoma progression via OX40/OX40L-mediated interaction with Th17 cells. Signal Transduct. Target. Ther..

[94] Olson E.N. (2014). MicroRNAs as therapeutic targets and biomarkers of cardiovascular disease. Sci. Transl. Med..

[95] Wang X., Ha T., Zou J., Ren D., Liu L., Zhang X., Kalbfleisch J., Gao X., Williams D., Li C. (2014). MicroRNA-125b protects against myocardial ischaemia/reperfusion injury via targeting p53-mediated apoptotic signalling and TRAF6. Cardiovasc. Res..

[96] Nanbo A., Katano H., Kataoka M., Hoshina S., Sekizuka T., Kuroda M., Ohba Y. (2018). Infection of epstein–barr virus in type III latency modulates biogenesis of exosomes and the expression profile of exosomal miRNAs in the burkitt lymphoma mutu cell lines. Cancers.

[97] Wen G., Zhou T., Gu W. (2021). The potential of using blood circular RNA as liquid biopsy biomarker for human diseases. Protein Cell.

[98] Hansen T.B., Jensen T.I., Clausen B.H., Bramsen J.B., Finsen B., Damgaard C.K., Kjems J. (2013). Natural RNA circles function as efficient microRNA sponges. Nature.

[99] Vo J.N., Cieslik M., Zhang Y., Shukla S., Xiao L., Zhang Y., Wu Y.-M., Dhanasekaran S.M., Engelke C.G., Cao X. (2019). The Landscape of Circular RNA in Cancer. Cell.

[100] Zheng X., Chen L., Zhou Y., Wang Q., Zheng Z., Xu B., Wu C., Zhou Q., Hu W., Wu C. (2019). A novel protein encoded by a circular RNA circPPP1R12A promotes tumor pathogenesis and metastasis of colon cancer via Hippo-YAP signaling. Mol. Cancer.

[101] He T., Tao W., Zhang L.-L., Wang B.-Y., Li K., Lu H.-M., Tang G.-J., He Y.-D., Li L.-Y. (2022). CircSCAF8 promotes growth and metastasis of prostate cancer through the circSCAF8-miR-140-3p/miR-335-LIF pathway. Cell Death Dis..

[102] van Zonneveld A.J., Kölling M., Bijkerk R., Lorenzen J.M. (2021). Circular RNAs in kidney disease and cancer. Nat. Rev. Nephrol..

[103] Yu C., Qian L., Uttamchandani M., Li L., Yao S.Q. (2015). Single-vehicular delivery of antagomir and small molecules to inhibit miR-122 function in hepatocellular carcinoma cells by using “smart” mesoporous silica nanoparticles. Angew. Chem. Int. Ed..

[104] Brock M., Samillan V.J., Trenkmann M., Schwarzwald C., Ulrich S., Gay R.E., Gassmann M., Ostergaard L., Gay S., Speich R. (2014). AntagomiR directed against miR-20a restores functional BMPR2 signalling and prevents vascular remodelling in hypoxia-induced pulmonary hypertension. Eur. Heart J..

[105] Krützfeldt J., Rajewsky N., Braich R., Rajeev K.G., Tuschl T., Manoharan M., Stoffel M. (2005). Silencing of microRNAs in vivo with ‘antagomirs’. Nature.

[106] Krützfeldt J., Kuwajima S., Braich R., Rajeev K.G., Pena J., Tuschl T., Manoharan M., Stoffel M. (2007). Specificity, duplex degradation and subcellular localization of antagomirs. Nucleic Acids Res..

[107] Ma L., Reinhardt F., Pan E., Soutschek J., Bhat B., Marcusson E.G., Teruya-Feldstein J., Bell G.W., Weinberg R.A. (2010). Therapeutic silencing of miR-10b inhibits metastasis in a mouse mammary tumor model. Nat. Biotechnol..

[108] Dereani S., Macor P., D’Agaro T., Mezzaroba N., Dal-Bo M., Capolla S., Zucchetto A., Tissino E., Del Poeta G., Zorzet S. (2014). Potential therapeutic role of antagomiR17 for the treatment of chronic lymphocytic leukemia. J. Hematol. Oncol..

[109] Hasan A., Khattab A., Islam M.A., Hweij K.A., Zeitouny J., Waters R., Sayegh M., Hossain M.M., Paul A. (2015). Injectable hydrogels for cardiac tissue repair after myocardial infarction. Adv. Sci..

[110] Andugulapati S.B., Gourishetti K., Tirunavalli S.K., Shaikh T.B., Sistla R. (2020). Biochanin-A ameliorates pulmonary fibrosis by suppressing the TGF-β mediated EMT, myofibroblasts differentiation and collagen deposition in in vitro and in vivo systems. Phytomedicine.

[111] Chen L., Wang Y., Pan Y., Zhang L., Shen C., Qin G., Ashraf M., Weintraub N., Ma G., Tang Y. (2013). Cardiac progenitor-derived exosomes protect ischemic myocardium from acute ischemia/reperfusion injury. Biochem. Biophys. Res. Commun..

[112] Santoso M.R., Ikeda G., Tada Y., Jung J.H., Vaskova E., Sierra R.G., Gati C., Goldstone A.B., von Bornstaedt D., Shukla P. (2020). Exosomes from induced pluripotent stem cell–derived cardiomyocytes promote autophagy for myocardial repair. J. Am. Heart Assoc..

[113] Wang Y., Zhang L., Li Y., Chen L., Wang X., Guo W., Zhang X., Qin G., He S.-h., Zimmerman A. (2015). Exosomes/microvesicles from induced pluripotent stem cells deliver cardioprotective miRNAs and prevent cardiomyocyte apoptosis in the ischemic myocardium. Int. J. Cardiol..

[114] Khan M., Nickoloff E., Abramova T., Johnson J., Verma S.K., Krishnamurthy P., Mackie A.R., Vaughan E., Garikipati V.N.S., Benedict C. (2015). Embryonic stem cell–derived exosomes promote endogenous repair mechanisms and enhance cardiac function following myocardial infarction. Circ. Res..

[115] Xuan L., Fu D., Zhen D., Wei C., Bai D., Yu L., Gong G. (2022). Extracellular vesicles derived from human bone marrow mesenchymal stem cells protect rats against acute myocardial infarction-induced heart failure. Cell Tissue Res..

[116] Rani S., Ryan A.E., Griffin M.D., Ritter T. (2015). Mesenchymal stem cell-derived extracellular vesicles: Toward cell-free therapeutic applications. Mol. Ther..

[117] Liu B., Lee B.W., Nakanishi K., Villasante A., Williamson R., Metz J., Kim J., Kanai M., Bi L., Brown K. (2018). Cardiac recovery via extended cell-free delivery of extracellular vesicles secreted by cardiomyocytes derived from induced pluripotent stem cells. Nat. Biomed. Eng..

[118] Sakai D., Nakamura Y., Nakai T., Mishima T., Kato S., Grad S., Alini M., Risbud M.V., Chan D., Cheah K.S.E. (2012). Exhaustion of nucleus pulposus progenitor cells with ageing and degeneration of the intervertebral disc. Nat. Commun..

[119] Binch A.L.A., Fitzgerald J.C., Growney E.A., Barry F. (2021). Cell-based strategies for IVD repair: Clinical progress and translational obstacles. Nat. Rev. Rheumatol..

[120] Moradi L., Vasei M., Dehghan M.M., Majidi M., Farzad Mohajeri S., Bonakdar S. (2017). Regeneration of meniscus tissue using adipose mesenchymal stem cells-chondrocytes co-culture on a hybrid scaffold: In vivo study. Biomaterials.

[121] Zhang Z.G., Buller B., Chopp M. (2019). Exosomes—Beyond stem cells for restorative therapy in stroke and neurological injury. Nat. Rev. Neurol..

[122] Shi Y., Li H., Chu D., Lin W., Wang X., Wu Y., Li K., Wang H., Li D., Xu Z. (2023). Rescuing nucleus pulposus cells from senescence via dual-functional greigite nanozyme to alleviate intervertebral disc degeneration. Adv. Sci..

[123] Novais E.J., Tran V.A., Johnston S.N., Darris K.R., Roupas A.J., Sessions G.A., Shapiro I.M., Diekman B.O., Risbud M.V. (2021). Long-term treatment with senolytic drugs Dasatinib and Quercetin ameliorates age-dependent intervertebral disc degeneration in mice. Nat. Commun..

[124] Feng C., Yang M., Lan M., Liu C., Zhang Y., Huang B., Liu H., Zhou Y. (2017). ROS: Crucial intermediators in the pathogenesis of intervertebral disc degeneration. Oxidative Med. Cell. Longev..

[125] Goldring S.R., Goldring M.B. (2016). Changes in the osteochondral unit during osteoarthritis: Structure, function and cartilage–bone crosstalk. Nat. Rev. Rheumatol..

[126] Lin C.-Y., Wang Y.-L., Chen Y.-J., Ho C.-T., Chi Y.-H., Chan L.Y., Chen G.-W., Hsu H.-C., Hwang D.W., Wu H.-C. (2022). Collagen-binding peptides for the enhanced imaging, lubrication and regeneration of osteoarthritic articular cartilage. Nat. Biomed. Eng..

[127] Oláh T., Reinhard J., Laschke M.W., Goebel L.K.H., Walter F., Schmitt G., Speicher-Mentges S., Menger M.D., Cucchiarini M., Pape D. (2022). Axial alignment is a critical regulator of knee osteoarthritis. Sci. Transl. Med..

[128] Muthu S., Korpershoek J.V., Novais E.J., Tawy G.F., Hollander A.P., Martin I. (2023). Failure of cartilage regeneration: Emerging hypotheses and related therapeutic strategies. Nat. Rev. Rheumatol..

[129] van der Kraan P.M. (2017). The changing role of TGFβ in healthy, ageing and osteoarthritic joints. Nat. Rev. Rheumatol..

[130] Richard D., Liu Z., Cao J., Kiapour A.M., Willen J., Yarlagadda S., Jagoda E., Kolachalama V.B., Sieker J.T., Chang G.H. (2020). Evolutionary selection and constraint on human knee chondrocyte regulation impacts osteoarthritis risk. Cell.

[131] Li H., Ghazanfari R., Zacharaki D., Lim H.C., Scheding S. (2016). Early growth response (EGR)-1 expression regulates colony forming capacity and hematopoietic support function in human primary bone marrow stromal stem cells. Blood.

[132] Doody K.M., Bottini N. (2016). Chondrocyte clocks make cartilage time-sensitive material. J. Clin. Investig..

[133] Hosseinzadeh A., Kamrava S.K., Joghataei M.T., Darabi R., Shakeri-Zadeh A., Shahriari M., Reiter R.J., Ghaznavi H., Mehrzadi S. (2016). Apoptosis signaling pathways in osteoarthritis and possible protective role of melatonin. J. Pineal Res..

[134] Tao S.-C., Huang J.-Y., Gao Y., Li Z.-X., Wei Z.-Y., Dawes H., Guo S.-C. (2021). Small extracellular vesicles in combination with sleep-related circRNA3503: A targeted therapeutic agent with injectable thermosensitive hydrogel to prevent osteoarthritis. Bioact. Mater..

[135] Das A., Segar C.E., Hughley B.B., Bowers D.T., Botchwey E.A. (2013). The promotion of mandibular defect healing by the targeting of S1P receptors and the recruitment of alternatively activated macrophages. Biomaterials.

[136] Raggatt L.J., Wullschleger M.E., Alexander K.A., Wu A.C.K., Millard S.M., Kaur S., Maugham M.L., Gregory L.S., Steck R., Pettit A.R. (2014). Fracture healing via periosteal callus formation requires macrophages for both initiation and progression of early endochondral ossification. Am. J. Pathol..

[137] Wang C., Inzana J.A., Mirando A.J., Ren Y., Liu Z., Shen J., O’Keefe R.J., Awad H.A., Hilton M.J. (2016). NOTCH signaling in skeletal progenitors is critical for fracture repair. J. Clin. Investig..

[138] Petite H., Viateau V., Bensaïd W., Meunier A., de Pollak C., Bourguignon M., Oudina K., Sedel L., Guillemin G. (2000). Tissue-engineered bone regeneration. Nat. Biotechnol..

[139] Grayson W.L., Bunnell B.A., Martin E., Frazier T., Hung B.P., Gimble J.M. (2015). Stromal cells and stem cells in clinical bone regeneration. Nat. Rev. Endocrinol..

[140] James A.W., LaChaud G., Shen J., Asatrian G., Nguyen V., Zhang X., Ting K., Soo C. (2016). A review of the clinical side effects of bone morphogenetic protein-2. Tissue Eng. Part B Rev..

[141] Wang X., Zou C., Hou C., Bian Z., Jiang W., Li M., Zhu L. (2023). Extracellular vesicles from bone marrow mesenchymal stem cells alleviate osteoporosis in mice through USP7-mediated YAP1 protein stability and the Wnt/β-catenin pathway. Biochem. Pharmacol..

[142] Duan J., Li H., Wang C., Yao J., Jin Y., Zhao J., Zhang Y., Liu M., Sun H. (2023). BMSC-derived extracellular vesicles promoted osteogenesis via Axin2 inhibition by delivering MiR-16-5p. Int. Immunopharmacol..

[143] Deng J., Wang X., Zhang W., Sun L., Han X., Tong X., Yu L., Ding J., Yu L., Liu Y. (2023). Versatile hypoxic extracellular vesicles laden in an injectable and bioactive hydrogel for accelerated bone regeneration. Adv. Funct. Mater..

[144] Ma Y., Sun L., Zhang J., Chiang C.-l., Pan J., Wang X., Kwak K.J., Li H., Zhao R., Rima X.Y. (2023). Exosomal mRNAs for angiogenic–osteogenic coupled bone repair. Adv. Sci..

[145] Xia W., Xie J., Cai Z., Liu X., Wen J., Cui Z.-K., Zhao R., Zhou X., Chen J., Mao X. (2021). Damaged brain accelerates bone healing by releasing small extracellular vesicles that target osteoprogenitors. Nat. Commun..

[146] Zheng Y., Ji S., Wu H., Tian S., Zhang Y., Wang L., Fang H., Luo P., Wang X., Hu X. (2017). Topical administration of cryopreserved living micronized amnion accelerates wound healing in diabetic mice by modulating local microenvironment. Biomaterials.

[147] Zhu Y., Zhang J., Song J., Yang J., Du Z., Zhao W., Guo H., Wen C., Li Q., Sui X. (2020). A multifunctional pro-healing zwitterionic hydrogel for simultaneous optical monitoring of pH and glucose in diabetic wound treatment. Adv. Funct. Mater..

[148] Sinwar P.D. (2015). The diabetic foot management—Recent advance. Int. J. Surg..

[149] Cho H., Blatchley M.R., Duh E.J., Gerecht S. (2019). Acellular and cellular approaches to improve diabetic wound healing. Adv. Drug Deliv. Rev..

[150] Patel S., Srivastava S., Singh M.R., Singh D. (2019). Mechanistic insight into diabetic wounds: Pathogenesis, molecular targets and treatment strategies to pace wound healing. Biomed. Pharmacother..

[151] Ning J., Zhao H., Chen B., Mi E.Z., Yang Z., Qing W., Lam K.W.J., Yi B., Chen Q., Gu J. (2019). Argon mitigates impaired wound healing process and enhances wound healing in vitro and in vivo. Theranostics.

[152] Kleiman A., Keats E.C., Chan N.G., Khan Z.A. (2012). Evolution of hemangioma endothelium. Exp. Mol. Pathol..

[153] Jiao J., Wang F., Huang J.-J., Huang J.-J., Li Z.-A., Kong Y., Zhang Z.-J. (2021). Microfluidic hollow fiber with improved stiffness repairs peripheral nerve injury through non-invasive electromagnetic induction and controlled release of NGF. Chem. Eng. J..

[154] Beggs S., Trang T., Salter M.W. (2012). P2X4R^+^ microglia drive neuropathic pain. Nat. Neurosci..

[155] Ichihara S., Inada Y., Nakamura T. (2008). Artificial nerve tubes and their application for repair of peripheral nerve injury: An update of current concepts. Injury.

[156] Dong M., Shi B., Liu D., Liu J.-H., Zhao D., Yu Z.-H., Shen X.-Q., Gan J.-M., Shi B.-l., Qiu Y. (2020). Conductive hydrogel for a photothermal-responsive stretchable artificial nerve and coalescing with a damaged peripheral nerve. ACS Nano.

[157] Ren H., Chen X., Tian M., Zhou J., Ouyang H., Zhang Z. (2018). Regulation of inflammatory cytokines for spinal cord injury repair through local delivery of therapeutic agents. Adv. Sci..

[158] Sykova E., Cizkova D., Kubinova S. (2021). Mesenchymal stem cells in treatment of spinal cord injury and amyotrophic lateral sclerosis. Front. Cell Dev. Biol..

[159] Veneruso V., Petillo E., Pizzetti F., Orro A., Comolli D., De Paola M., Verrillo A., Baggiolini A., Votano S., Castiglione F. (2023). Synergistic pharmacological therapy to modulate glial cells in spinal cord injury. Adv. Mater..

[160] Burnstine-Townley A., Eshel Y., Amdursky N. (2020). Conductive scaffolds for cardiac and neuronal tissue engineering: Governing factors and mechanisms. Adv. Funct. Mater..

[161] Qian Y., Zhao X., Han Q., Chen W., Li H., Yuan W. (2018). An integrated multi-layer 3D-fabrication of PDA/RGD coated graphene loaded PCL nanoscaffold for peripheral nerve restoration. Nat. Commun..

[162] Xiao Y., Hu X., Jiang P., Qi Z. (2023). Thermos-responsive hydrogel system encapsulated engineered exosomes attenuate inflammation and oxidative damage in acute spinal cord injury. Front. Bioeng. Biotechnol..

[163] Fan L., Liu C., Chen X., Zheng L., Zou Y., Wen H., Guan P., Lu F., Luo Y., Tan G. (2022). Exosomes-loaded electroconductive hydrogel synergistically promotes tissue repair after spinal cord injury via immunoregulation and enhancement of myelinated axon growth. Adv. Sci..

[164] Kelly P.N. (2018). The cancer immunotherapy revolution. Science.

[165] Duan Z., Luo Y. (2021). Targeting macrophages in cancer immunotherapy. Signal Transduct. Target. Ther..

[166] Banchereau J., Palucka K. (2018). Cancer vaccines on the move. Nat. Rev. Clin. Oncol..

[167] Waldman A.D., Fritz J.M., Lenardo M.J. (2020). A guide to cancer immunotherapy: From T cell basic science to clinical practice. Nat. Rev. Immunol..

[168] Ji P., Sun W., Zhang S., Xing Y., Wang C., Wei M., Li Q., Ji G., Yang G. (2023). Modular Hydrogel Vaccine for Programmable and Coordinate Elicitation of Cancer Immunotherapy. Adv. Sci..

[169] Palla A.R., Ravichandran M., Wang Y.X., Alexandrova L., Yang A.V., Kraft P., Holbrook C.A., Schürch C.M., Ho A.T.V., Blau H.M. (2021). Inhibition of prostaglandin-degrading enzyme 15-PGDH rejuvenates aged muscle mass and strength. Science.

[170] Cohen S., Nathan J.A., Goldberg A.L. (2015). Muscle wasting in disease: Molecular mechanisms and promising therapies. Nat. Rev. Drug Discov..

[171] Gutiérrez-Pérez P., Santillán E.M., Lendl T., Wang J., Schrempf A., Steinacker T.L., Asparuhova M., Brandstetter M., Haselbach D., Cochella L. (2021). miR-1 sustains muscle physiology by controlling V-ATPase complex assembly. Sci. Adv..

[172] Coenen-Stass A.M.L., Wood M.J.A., Roberts T.C. (2017). Biomarker potential of extracellular miRNAs in duchenne muscular dystrophy. Trends Mol. Med..

[173] Li J., Chan M.C., Yu Y., Bei Y., Chen P., Zhou Q., Cheng L., Chen L., Ziegler O., Rowe G.C. (2017). miR-29b contributes to multiple types of muscle atrophy. Nat. Commun..

[174] Yu Y., Li X., Liu L., Chai J., Haijun Z., Chu W., Yin H., Ma L., Duan H., Xiao M. (2016). miR-628 promotes burn-induced skeletal muscle atrophy via targeting irs1. Int. J. Biol. Sci..

[175] Dai H., Luo J., Deng L., Song C., Deng Z., Wu Y., Gu S., Xu J. (2023). Hierarchically injectable hydrogel sequentially delivers antagomiR-467a-3p-loaded and antagomiR-874-5p-loaded satellite-cell-targeting bioengineered extracellular vesicles attenuating sarcopenia. Adv. Healthc. Mater..

[176] Alizzi A.M., Summers P., Boon V.H., Tantiongco J.-P., Thompson T., Leslie B.J., Williams D., Steele M., Bidstrup B.P., Diqer A.-M.A. (2012). Reduction of Post-surgical Pericardial Adhesions Using a Pig Model. Heart Lung Circ..

[177] Fujita M., Policastro G.M., Burdick A., Lam H.T., Ungerleider J.L., Braden R.L., Huang D., Osborn K.G., Omens J.H., Madani M.M. (2021). Preventing post-surgical cardiac adhesions with a catechol-functionalized oxime hydrogel. Nat. Commun..

[178] Stapleton L.M., Steele A.N., Wang H., Lopez Hernandez H., Yu A.C., Paulsen M.J., Smith A.A.A., Roth G.A., Thakore A.D., Lucian H.J. (2019). Use of a supramolecular polymeric hydrogel as an effective post-operative pericardial adhesion barrier. Nat. Biomed. Eng..

[179] Ferraris V.A. (2018). Pericardial adhesions and cardiac surgeons’ nightmares. J. Thorac. Cardiovasc. Surg..

[180] Wang L., Chen P., Pan Y., Wang Z., Xu J., Wu X., Yang Q., Long M., Liu S., Huang W. (2023). Injectable photocurable Janus hydrogel delivering hiPSC cardiomyocyte-derived exosome for post–heart surgery adhesion reduction. Sci. Adv..

[181] Wu X., Guo W., Wang L., Xu Y., Wang Z., Yang Y., Yu L., Huang J., Li Y., Zhang H. (2022). An Injectable Asymmetric-Adhesive Hydrogel as a GATA6^+^ Cavity Macrophage Trap to Prevent the Formation of Postoperative Adhesions after Minimally Invasive Surgery. Adv. Funct. Mater..

[182] Susantitaphong P., Cruz D.N., Cerda J., Abulfaraj M., Alqahtani F., Koulouridis I., Jaber B.L. (2013). World incidence of aki: A meta-analysis. Clin. J. Am. Soc. Nephrol..

[183] Bulluck H., Hausenloy D.J. (2018). Modulating NAD^+^ metabolism to prevent acute kidney injury. Nat. Med..

[184] Nilforoushzadeh M.A., Zare S., Farshi S., Mahmoudbeyk M., Nouri M., Jaffary F., Nikkhah N. (2021). Clinical, biometric, and ultrasound assessment of the effects of the autologous fibroblast cells transplantation on nasolabial fold wrinkles. J. Cosmet. Dermatol..

[185] Xing M., Liu H., Meng F., Ma Y., Zhang S., Gao Y. (2022). Design and evaluation of complex polypeptide-loaded dissolving microneedles for improving facial wrinkles in different areas. Polymers.

[186] Tezel A., Fredrickson G.H. (2008). The science of hyaluronic acid dermal fillers. J. Cosmet. Laser Ther..

[187] You D.G., An J.Y., Um W., Jung J.M., Oh B.H., Nguyen V.Q., Jeon J., Lee J., Jo D.-G., Cho Y.W. (2022). Stem cell-derived extracellular vesicle-bearing dermal filler ameliorates the dermis microenvironment by supporting CD301b-expressing macrophages. ACS Nano.

[188] Wang J., Li X., Wang S., Cui J., Ren X., Su J. (2023). Bone-targeted exosomes: Strategies and applications. Adv. Healthc. Mater..

[189] Liu H., Zhang Q., Wang S., Weng W., Jing Y., Su J. (2022). Bacterial extracellular vesicles as bioactive nanocarriers for drug delivery: Advances and perspectives. Bioact. Mater..

[190] Ofir-Birin Y., Regev-Rudzki N. (2019). Extracellular vesicles in parasite survival. Science.

[191] Zonneveld M.I., van Herwijnen M.J.C., Fernandez-Gutierrez M.M., Giovanazzi A., de Groot A.M., Kleinjan M., van Capel T.M.M., Sijts A.J.A.M., Taams L.S., Garssen J. (2021). Human milk extracellular vesicles target nodes in interconnected signalling pathways that enhance oral epithelial barrier function and dampen immune responses. J. Extracell. Vesicles.

[192] Liu W., Zhang X., Jiang X., Dai B., Zhang L., Zhu Y. (2024). Decellularized extracellular matrix materials for treatment of ischemic cardiomyopathy. Bioact. Mater..

[193] Phan V.H.G., Duong H.-S., Le Q.-G.T., Janarthanan G., Vijayavenkataraman S., Nguyen H.-N.H., Nguyen B.-P.T., Manivasagan P., Jang E.-S., Li Y. (2023). Nanoengineered injectable hydrogels derived from layered double hydroxides and alginate for sustained release of protein therapeutics in tissue engineering applications. J. Nanobiotechnol..

[194] Mardpour S., Ghanian M.H., Sadeghi-abandansari H., Mardpour S., Nazari A., Shekari F., Baharvand H. (2019). Hydrogel-mediated sustained systemic delivery of mesenchymal stem cell-derived extracellular vesicles improves hepatic regeneration in chronic liver failure. ACS Appl. Mater. Interfaces.

[195] Wei Y., Shi M., Zhang J., Zhang X., Shen K., Wang R., Miron R.J., Xiao Y., Zhang Y. (2020). Autologous versatile vesicles-incorporated biomimetic extracellular matrix induces biomineralization. Adv. Funct. Mater..

[196] Zhou Y., Liu S., Zhao M., Wang C., Li L., Yuan Y., Li L., Liao G., Bresette W., Zhang J. (2019). Injectable extracellular vesicle-released self-assembling peptide nanofiber hydrogel as an enhanced cell-free therapy for tissue regeneration. J. Control. Release.

[197] Xiong Y., Chen L., Liu P., Yu T., Lin C., Yan C., Hu Y., Zhou W., Sun Y., Panayi A.C. (2022). All-in-one: Multifunctional hydrogel accelerates oxidative diabetic wound healing through timed-release of exosome and fibroblast growth factor. Small.

[198] Bao Z., Xian C., Yuan Q., Liu G., Wu J. (2019). Natural Polymer-Based Hydrogels with Enhanced Mechanical Performances: Preparation, Structure, and Property. Adv. Healthc. Mater..

[199] Sanmartín-Masiá E., Poveda-Reyes S., Gallego Ferrer G. (2017). Extracellular matrix–inspired gelatin/hyaluronic acid injectable hydrogels. Int. J. Polym. Mater. Polym. Biomater..

[200] Chen J., Xu X., Liu M., Li Y., Yu D., Lu Y., Xiong M., Wyman I., Xu X., Wu X. (2021). Topological cyclodextrin nanoparticles as crosslinkers for self-healing tough hydrogels as strain sensors. Carbohydr. Polym..

[201] Cao W., Zhou X., Tu C., Wang Z., Liu X., Kang Y., Wang J., Deng L., Zhou T., Gao C. (2023). A broad-spectrum antibacterial and tough hydrogel dressing accelerates healing of infected wound in vivo. Biomater. Adv..

[202] Forteza-Genestra M.A., Antich-Rosselló M., Calvo J., Gayà A., Monjo M., Ramis J.M. (2020). Purity Determines the Effect of Extracellular Vesicles Derived from Mesenchymal Stromal Cells. Cells.

[203] Garcia-Motta H., Bonifacio M., Martignago C.C.S., Souza-Silva L.C., Soares-Silva B., Parisi J.R., Assis L., Ribeiro D.A., Ribeiro A.M., Rennó A.C. (2023). Hydrogels loaded with mesenchymal stem cells extracellular vesicles for treating knee joint disorders: A systematic review. Regen. Eng. Transl. Med..

[204] Trenkenschuh E., Richter M., Heinrich E., Koch M., Fuhrmann G., Friess W. (2022). Enhancing the stabilization potential of lyophilization for extracellular vesicles. Adv. Healthc. Mater..

[205] Li Q., Gong S., Yao W., Yang Z., Wang R., Yu Z., Wei M. (2021). Exosome loaded genipin crosslinked hydrogel facilitates full thickness cutaneous wound healing in rat animal model. Drug Deliv..

[206] Buwalda S.J., Vermonden T., Hennink W.E. (2017). Hydrogels for therapeutic delivery: Current developments and future directions. Biomacromolecules.

[207] Nguyen M.K., Jeon O., Dang P.N., Huynh C.T., Varghai D., Riazi H., McMillan A., Herberg S., Alsberg E. (2018). RNA interfering molecule delivery from in situ forming biodegradable hydrogels for enhancement of bone formation in rat calvarial bone defects. Acta Biomater..

[208] Yao X., Zhu G., Zhu P., Ma J., Chen W., Liu Z., Kong T. (2020). Omniphobic zif-8@hydrogel membrane by microfluidic-emulsion-templating method for wound healing. Adv. Funct. Mater..

[209] Liu T., Du Y., Yan Y., Song S., Qi J., Xia X., Hu X., Chen Q., Liu J., Zeng X. (2023). pH-responsive dual-functional hydrogel integrating localized delivery and anti-cancer activities for highly effective therapy in PDX of OSCC. Mater. Today.

[210] Wang L.L., Chung J.J., Li E.C., Uman S., Atluri P., Burdick J.A. (2018). Injectable and protease-degradable hydrogel for siRNA sequestration and triggered delivery to the heart. J. Control. Release.

[211] Han B.H., Kim S., Seo G., Heo Y., Chung S., Kang J.Y. (2020). Isolation of extracellular vesicles from small volumes of plasma using a microfluidic aqueous two-phase system. Lab A Chip.

[212] Chen W., Zhu Y., Liu R., Kong B., Xia N., Zhao Y., Sun L. (2023). Screening Therapeutic Effects of MSC-EVs to Acute Lung Injury Model on A Chip. Adv. Healthc. Mater..

[213] Li Z., Liu C., Cheng Y., Li Y., Deng J., Bai L., Qin L., Mei H., Zeng M., Tian F. (2023). Cascaded microfluidic circuits for pulsatile filtration of extracellular vesicles from whole blood for early cancer diagnosis. Sci. Adv..

[214] Gao Y., Peng K., Mitragotri S. (2021). Covalently crosslinked hydrogels via step-growth reactions: Crosslinking chemistries, polymers, and clinical impact. Adv. Mater..

[215] Yuan L., Wu Y., Gu Q.-s., El-Hamshary H., El-Newehy M., Mo X. (2017). Injectable photo crosslinked enhanced double-network hydrogels from modified sodium alginate and gelatin. Int. J. Biol. Macromol..

[216] Kim S., Healy K.E. (2003). Synthesis and characterization of injectable poly(n-isopropylacrylamide-co-acrylic acid) hydrogels with proteolytically degradable cross-links. Biomacromolecules.

[217] Zhang J., Wu J., Wang G., He L., Zheng Z., Wu M., Zhang Y. (2023). Extracellular vesicles: Techniques and biomedical applications related to single vesicle analysis. ACS Nano.

[218] Ji Q., Zhou L., Sui H., Yang L., Wu X., Song Q., Jia R., Li R., Sun J., Wang Z. (2020). Primary tumors release ITGBL1-rich extracellular vesicles to promote distal metastatic tumor growth through fibroblast-niche formation. Nat. Commun..

